# Impact of Maternal Overweight and Obesity on Pregnancy Outcomes Following Cesarean Delivery: A Retrospective Cohort Study

**DOI:** 10.3390/healthcare13151893

**Published:** 2025-08-02

**Authors:** Zlatina Nikolova, Milena Sandeva, Ekaterina Uchikova, Angelina Kirkova-Bogdanova, Daniela Taneva, Marieta Vladimirova, Lyubomira Georgieva

**Affiliations:** 1 Department of Midwifery Care, Faculty of Public Health, Medical University—Plovdiv, 4002 Plovdiv, Bulgaria; zlatina.nikolova@mu-plovdiv.bg (Z.N.); milena.sandeva@mu-plovdiv.bg (M.S.); marieta.vladimirova@mu-plovdiv.bg (M.V.); lyubomira.georgieva@mu-plovdiv.bg (L.G.); 2 Medical Simulation Training Centre, Medical Faculty, Medical University—Plovdiv, 4002 Plovdiv, Bulgaria; 3 Department of Obstetrics and Gynaecology, Medical Faculty, Medical University—Plovdiv, 4002 Plovdiv, Bulgaria; ekaterina.uchikova@mu-plovdiv.bg; 4 Clinic of Obstetrics and Gynecology, University Hospital “St. George”, 4000 Plovdiv, Bulgaria; 5 Department of Medical Informatics, Biostatistics and E-Learning, Faculty of Public Health, Medical University—Plovdiv, 4002 Plovdiv, Bulgaria; 6 Department of Nursing Care, Faculty of Public Health, Medical University—Plovdiv, 4004 Plovdiv, Bulgaria; daniela.taneva@mu-plovdiv.bg

**Keywords:** maternal obesity, cesarean section, pregnancy outcome, complications, pregnancy-induced hypertension, hypertensive disorders

## Abstract

**Background/Objectives**: Maternal overweight and obesity are critical factors increasing the risk of various pregnancy complications. Maternal obesity can lead to fetal macrosomia and a heightened risk of intrauterine death, with long-term implications for the child’s health. This study aimed to analyze the incidence of obesity and its impact on pregnancy outcomes in women who delivered by cesarean section at the University Hospital “St. George”, Plovdiv. **Methods**: A single-center retrospective cohort study was conducted. The documentary method was used for gathering data. Records were randomly selected. The statistical methods used included mean values, confidence intervals (of mean), frequency, and the Kolmogorov–Smirnov test for normality of distribution. Data comparisons were performed using the Mann–Whitney test. Mean values of numerical variables were compared using the independent samples *t*-test. **Results**: In total, 46.36% of women in this study were affected by obesity to varying degrees, and the proportion of women who were overweight at the end of their pregnancy was 37.85%. In the studied cohort, 15.99% of women were affected by hypertensive complications. This significant prevalence of obesity highlights concerns regarding body weight among women of reproductive age. This study emphasized a strong correlation between maternal obesity, particularly severe obesity, and the occurrence of preeclampsia. **Conclusions**: In this study among women who delivered by cesarean section, a significant proportion of them were affected by overweight and obesity. Data for our country are insufficient, and a more in-depth study of this problem is needed. Future research should explore the long-term impacts of maternal obesity on the health of the mother and the newborn.

## 1. Introduction

Maternal overweight and obesity significantly increase the risk of complications during pregnancy, labor, and the perinatal period for the child. Additionally, excessive weight gain during pregnancy and the retention of weight postpartum can negatively impact future fertility and subsequent pregnancies [1]. Baseline (preconception) weight and moderate gestational weight gain (GWG) are crucial factors for the successful progression of pregnancy. Obesity is linked to a markedly increased risk of developing gestational diabetes, pregnancy-induced hypertension, preeclampsia, venous thromboembolism, and cesarean delivery [2]. For the fetus, maternal obesity is associated with conditions such as fetal macrosomia and a heightened risk of intrauterine death, as well as an increased incidence of obesity and metabolic syndrome in childhood. These effects may result from altered placental function associated with maternal obesity, although the underlying mechanisms remain unclear [3]. Furthermore, maternal obesity has the potential to perpetuate a cycle of obesity and insulin resistance across generations [4].

The available data for the Bulgarian population indicate that 54.7% of adults (over 19 years old) were affected by overweight or obesity in 2020 [5]. Unfortunately, our targeted search failed to find accurate data on obese women of reproductive age or pregnant women in our country. Also, annual data on the level of overweight and obesity in the period after 2020 are not available for our country.

**Aim:** This study aimed to analyze the incidence of obesity and its impact on pregnancy outcomes in women who delivered by cesarean section (CS) from 2021 to 2023 at the “Clinic of Obstetrics and Gynaecology” of the University Hospital “St. George”, Plovdiv. The outcome of the pregnancy includes the results for the mother and the newborn. For the mother, comorbidities and pregnancy complications, specifically hypertensive complications, were included in this study. For the newborn, we analyzed pregnancy outcomes such as prematurity, gestational age, birth weight, and Apgar score.

## 2. Materials and Methods

A retrospective study was conducted over a three-year period (May 2021–May 2023) at the “Clinic of Obstetrics and Gynaecology” of the University Hospital “St. George”, Plovdiv. A documentary method was employed to gather data from hospital records, specifically the “Pregnancy and Birth History” of the standardized patient records of women discharged from the clinic following giving birth via cesarean section. Women were selected randomly. The patients’ medical records are on paper. In order to comply with the principle of randomness, data were collected from one of every fifth medical record in the indicated cohort. The inclusion criteria were as follows: (1) the patient underwent delivery via CS; (2) complete data from the woman’s medical history at admission and relevant anthropometric measurements were available. Files with missing data were excluded from the analysis. The key information collected included age, parity, height and weight, body mass index (BMI), abdominal circumference, concomitant diseases, presence of arterial hypertension (AH) or gestational diabetes mellitus (GDM), indications for CS, and complications during pregnancy and childbirth. The primary data collection was conducted by a physician and a midwife during the admission of the woman to the clinic. Data on concomitant diseases were collected from previous medical documentation, and in the cases of women admitted without such, the diseases were self-reported by the patients. All of the anthropometric measurements of the mother and the newborn were taken by a specialist during the diagnostic and treatment process. BMI was calculated by a medical professional based on the participants’ measured height and weight.

### 2.1. Data Analysis

In this study, descriptive statistical analysis methods were used, namely relative shares and the mean. The normality of the distribution of the variables was tested using the Kolmogorov–Smirnov test. Data comparisons were made using the Mann–Whitney test. The central tendencies of the numerical values are represented by the mean, confidence intervals (CIs) of the mean, and standard deviation (SD). The mean values of the numerical variables were compared using the independent samples *t*-Test. Statistically significant differences were accepted when the *p*-value was lower than 0.05. Statistical analysis was performed using SPSS version 23.0 software (SPSS Inc., Chicago, IL, USA).

### 2.2. Ethical Approval

This study was approved by the Ethical Committee of the Medical University of Plovdiv, with meeting protocol No 1/05 Feb 2025.

## 3. Results

Based on the established inclusion criteria, 494 cases of women following giving birth via a CS were included in the study sample. The mean age of the participants was approximately 29.33 years (95% CI 28.79–29.93), with the youngest patient being 14 years old and the oldest being 48 years. Singleton pregnancies constituted 96.56% of the entire cohort, or 477 women, and there were 17 twin pregnancies (3.44%). Among the women, 91.30% (451) were in their tenth lunar month of pregnancy, while 6.28% (31) were in their ninth lunar month, 1.62% in their eighth month, and 0.81% in their seventh month. The key characteristics of the sample are summarized in Table 1.

Pregnancy was complicated by diabetes (either gestational diabetes mellitus or previous diabetes) in 4.25% (N = 21) of the women included in this study.

The analysis of pregnancy outcomes revealed that 51.82% of the women underwent an emergency CS, while 48.18% underwent planned procedures. The primary reason for performing a CS was a previous cesarean, accounting for 37.45% of the cases. Other indications for surgical intervention included moderate preeclampsia in 9.31% of the cases (46 women), severe preeclampsia in 2.32% of the cases (11 women), abruptio placentae in 1.21% of the cases (6 women), a large fetus in 6.07% of the cases (30 cases), intrauterine growth restriction (IUGR) in 1.82% of the cases (9 cases), polyhydramnios in 1.21% of the cases (6 women), blood pressure values unresponsive to therapy in 0.81% of the cases (4 women), and obesity in one woman (0.20%). GDM was noted as an indication in 2.43% of the cases (12 women) and diabetic fetopathy in 0.4% of the cases (2 women). It is important to emphasize here that often the accompanying diseases registered in the medical documentation are not a direct reason for performing a CS. Some of the accompanying diseases may represent a secondary or tertiary indication for CS. For this reason, there is a discrepancy between the accompanying diseases presented in Table 1 and the reasons for performing a CS.

It is notable that only one woman was classified as having “obesity” among the reasons for surgical intervention. However, the overall data indicate that the total number of patients with varying degrees of obesity (I, II, or III) is 229, yet only 1 of these patients had obesity cited as a reason for the surgical procedure. Obesity is not a standalone indication for a cesarean section. It is indicated as a secondary or tertiary indication for abdominal delivery. More frequently occurring complications of overweight and obesity are indications for surgical intervention. It is crucial to highlight that this significant discrepancy between the stated reasons for CS and the actual obese women suggests potential underreporting or normalization of obesity by healthcare professionals.

The analyses indicate that the data concerning the women’s age, weight at admission, and systolic and diastolic arterial pressure do not follow a normal distribution, whereas the weights of the newborns are normally distributed.

**Figure 1:**
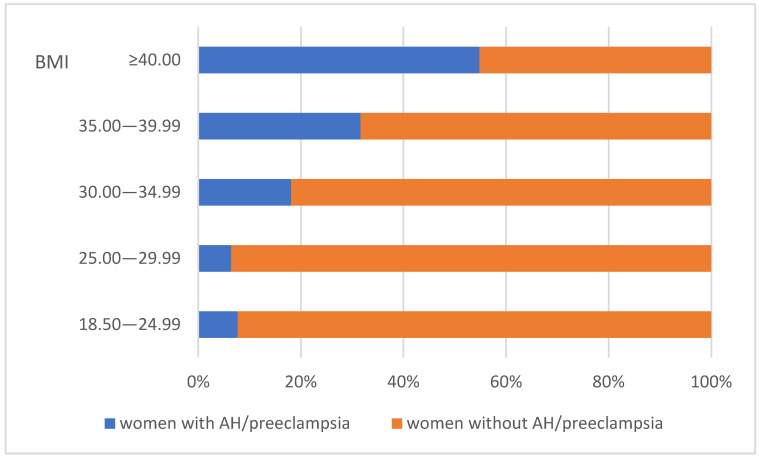
Relative shares of women in the study affected by AH/preeclampsia by BMI classes. Abbreviations: BMI—body mass index; AH—arterial hypertension.

The results indicate that a total of 79 (15.99% of the whole cohort) women experienced hypertensive disorders, both pre- and post-pregnancy. Out of these, 64 received treatment, while 15 did not. The conditions identified included preeclampsia (both moderate and severe), pre-existing arterial hypertension (pre-AH), and hypertension with superimposed preeclampsia. Among the patients diagnosed with hypertension, the average weight upon admission was 95.16 ± 21.11 kg (95% CI 90.46–99.92), whereas in the uncomplicated group, the mean weight was lower—78.91 ± 14.71 kg (95% CI 77.49–80.33). A statistically significant difference in body weight between the two groups was observed (*p* < 0.001). Additionally, as anticipated, there was a considerable difference in both the systolic and diastolic blood pressure measurements between the two groups (*p* < 0.001), although the majority of those affected (64 or 81.01%) were treated, leading to the recorded values being influenced by antihypertensive therapy. This study reveals a distinct trend indicating an increased incidence of hypertension and preeclampsia among obese women. In the cohort with a BMI over 30.00, 26.64% were affected, compared to only 6.79% in the group with a BMI below 30.00. Figure 1 illustrates the distribution of women affected by hypertensive disorders based on BMI. The number of patients with AH or preeclampsia is shown alongside the unaffected patients. The trend of increasing proportions of hypertension and preeclampsia cases is clear across the three classes of obesity.

Divided into two groups—those with obesity (in all degrees) and those without—we found that the mean systolic pressure values were 122.49 ± 17.71 mmHg (95% CI 120.18–124.79) and 114.18 ± 13.32 mmHg (95% CI 112.57–115.80), respectively. In terms of diastolic pressure, the mean values for both groups of women were 76.73 ± 13.14 mmHg (95% CI 75.02–78.45) and 70.82 ± 9.54 mmHg (95% CI 69.67–71.98), respectively. The statistical analysis reveals a significant difference between the obese and non-obese groups for both measurements (*p* < 0.001).

The mean weight of the newborns in grams is 3091.94 ± 553.07. Among the sample, full-term newborns constitute 87.67% (448), while 12.33% (63) are classified as premature. The distribution of the children according to degrees of prematurity is detailed in Table 2.

The comparative analysis of neonatal weights derived from singleton pregnancies among women with and without hypertensive disorders revealed a statistically significant disparity in the mean values (*p* = 0.001). Here, we excluded children from twin pregnancies as they have a high incidence of low birth weight. The average weight of the newborns of women affected by hypertensive disorders was recorded at 2940.66 g (95% CI 2785.88—3095.44), in contrast to 3172.54 g (95% CI 3123.32—3221.77) for those without such disorders. A notable increase in the risk of preterm delivery was observed among women diagnosed with AH or preeclampsia, with an odds ratio of 3.98 (95% CI 2.08–7.63), indicating an almost fourfold increase in probability. The incidence of premature births among this cohort stands at 23.68%, compared to only 7.23% in the group without AH (*p* < 0.001), further highlighting the implications of gestational hypertension on neonatal outcomes.

An examination of maternal age as a contributing factor to the prevalence of AH/preeclampsia revealed an escalating incidence in mothers over 35 years. The prevalence among women aged 34 years and younger is 14.17%, whereas it escalates to 22.92% in those aged over 35, with these differences established as statistically significant (*p* = 0.037). In refining the analysis, twin pregnancies were also excluded to ensure that the focus remained on singleton births.

There was a statistically significant difference in the gestational week of delivery between the two groups of women (*p* = 0.001). In women who gave birth with AH/preeclampsia, the average gestational week at delivery was 36.82 ± 2.25 (95% CI 36.32–37.33), while for those without hypertensive disorders, it was 38.17 ± 1.58 weeks (95% CI 38.02–38.32). These results align with our expectations, considering the complications associated with gestational hypertension and preeclampsia, which often necessitate preterm delivery to ensure the health of the mother and fetus. Here, we excluded children from twin pregnancies as they have a high incidence of preterm births.

When evaluating neonatal weight data, no significant differences were noted within the subset of mothers suffering from diabetes/GDM (*p* = 0.702). It is important to note that our sample comprised only 21 women diagnosed with diabetes, which could lead to certain biases. However, a statistically significant disparity was observed in the Apgar scores at both the 1 min and 5 min marks across both maternal groups, with and without diabetes. The tests revealed *p*-values of *p* = 0.020 (1st minute) and *p* < 0.001 (5th minute).

## 4. Discussion

The prevalence of overweight and obesity among women of reproductive age has risen significantly over the past three decades, leading to an increasing number of pregnancy complications. These complications include GDM, arterial hypertension, preeclampsia, IUGR, labor obstructions, late fetal death, and stillbirth [1]. Our study revealed a prevalence of 37.85% for overweight and 46.36% for obesity in the cohort of women undergoing CS, cumulatively accounting for 84.21% of the participants. Globally, the prevalence of overweight and obesity in pregnant women has reached epidemic proportions, with estimates suggesting that over 25% of all women worldwide may be obese by 2025. The reported pre-pregnancy rates across Europe typically range between 26.8% and 54% [6]. According to Lourenço J. and Guedes-Martins L., the global prevalence of overweight and obesity in pregnant women is around 50% [7]. The substantial difference from general European and global statistics points to a particularly severe public health situation within the studied Bulgarian population. This highlights a critical gap in national data and underscores the need for more targeted national surveillance and intervention strategies.

The literature confirms that maternal obesity independently confers a risk of obesity and metabolic syndrome to the child [6]. These intergenerational effects often stem from epigenetic programming and altered metabolic pathways during fetal development, leading to long-term public health consequences that extend far beyond the immediate perinatal period [8]. This broader implication underscores the need for comprehensive preconception and antenatal interventions to break this cycle.

Our study observed an increased prevalence of hypertensive disorders in obese women. In the cohort with a BMI above 30.00, the incidence of women with hypertensive disorders was 26.64%, while in those with a BMI below 30.00, the proportion was 6.79%. The recent literature consistently confirms a linear association between increasing pre-pregnancy BMI and the risk of preeclampsia and gestational hypertension. The underlying pathophysiology is complex, involving a state of poor maternal cardiometabolic health [7].

Hypertensive disorders of pregnancy are a leading cause of perinatal adverse outcomes, and complications occur predominantly in low- and middle-income countries [9]. The present study’s finding of an almost fourfold increased risk of preterm delivery among women diagnosed with AH or preeclampsia is an important observation that aligns with the broader literature [10]. This emphasizes the immediate and severe neonatal consequences associated with maternal hypertensive complications, highlighting the need for vigilant monitoring and timely intervention in obese pregnant women. Hypertensive disorders of pregnancy are strongly associated with increased risks of preterm birth, low birth weight, and fetal macrosomia [10].

The average GWG during pregnancy in the studied cohort was 15.06 kg, with increasing rates across obesity classes. While our study noted no significant difference in neonatal weights for mothers with diabetes or GDM, we acknowledged the small sample size for this subgroup as a potential limitation. This observation, however, opens up a discussion point: the role of GWG as a distinct pregnancy risk factor. Excessive GWG is a significant modifiable risk factor independently associated with adverse outcomes, including gestational hypertension, emergency CS, and large-for-gestational-age infants [11]. Recent research suggests that the optimal GWG ranges for women with obesity should be lower than the current general recommendations, varying by the severity of the pre-pregnancy BMI [11]. For instance, the optimal GWG for women with BMI ≥ 50 kg/m^2^ may be as low as >0 to ≤8 kg [12], and for those with GDM, even negative weight gain (−7 to 1 kg) might be optimal for obese women [11]. Conversely, GWG below the recommended level can increase the risk of small-for-gestational-age (SGA) infants [11].

The emerging consensus for tailored GWG guidelines based on the severity of obesity represents a significant evolution in clinical practice [12]. Our current study provides data that will be valuable for similar specialized analyses in future studies within the Bulgarian context.

Despite 46.36% of our cohort being obese, only one woman had “obesity” listed as a reason for her giving birth via cesarean section. This observation of underreporting may not be an isolated incident but rather reflects a broader, systemic problem of weight bias and stigma in healthcare settings [13]. Such biases can lead to reduced quality of care, disrespectful communication, and assumptions that all health problems are attributable to weight. Furthermore, healthcare professionals often receive insufficient education on obesity [13].

Obesity is a well-established risk factor for cesarean delivery, with the rates increasing linearly with BMI [6]. Common indications for CS in obese women include failure to progress in labor and cephalopelvic disproportion [14]. The persistence of high CS rates in obese patients even with standardized labor induction protocols [15] suggests that underlying physiological differences related to obesity (e.g., altered labor progression) are also significant contributors to the increased need for CS. This implies that this cohort of women requires a multifaceted approach, as well as an understanding and management of the unique physiological challenges associated with labor and obesity.

Chang et al. [16] note that many studies associate advanced maternal age (specifically, women over 35 years) with higher incidences of preeclampsia and other complications. Our findings align with this observation regarding hypertensive complications; however, due to the relatively small sample size of older women in our study, we must interpret these results with caution. A larger cohort study is necessary to draw more definitive conclusions for our population. Similarly, Martin-Alonso et al. [17] suggest a correlation between high BMI and an increased risk of GDM, but the limited number of cases in our study prevented us from making a meaningful comparison.

We believe that our findings contribute to the limited national data available in Bulgaria on maternal obesity and its outcomes. The absence of socioeconomic or behavioral data in our study, due to the retrospective design, limits interpretation but also highlights areas for future prospective research. The inclusion of these variables is essential, as social determinants of health significantly mediate both obesity prevalence and pregnancy outcomes.

### 4.1. Limitations of the Study

This study has several limitations that should be acknowledged. First, the retrospective design restricts the ability to establish causal relationships, as the data were collected from existing medical records rather than through prospective observation. Consequently, missing or incomplete data may have affected the findings.

Second, although we aimed to ensure the accurate recording of cesarean section indications, some contributing factors—such as obesity—may be underreported or clinically normalized. For example, only 1 out of 229 women with obesity had obesity listed as a primary indication for cesarean delivery, which may indicate underreporting or bias in documentation.

Third, important potential confounders such as smoking status, socioeconomic background, dietary habits, and other harmful health behaviors (e.g., alcohol use, physical inactivity) were not assessed or recorded in the dataset. They were not included in the medical records, as there was no requirement to do so. Their absence limits the ability to adjust for these variables, which may have influenced both the maternal outcomes and clinical decisions.

Fourth, this study was conducted at a single institution, which may affect the generalizability of the results to other regions, healthcare systems, or population groups.

Finally, there is a possibility of selection bias due to the inclusion criteria and the nature of the available data. Future research should include prospective, multicenter studies with comprehensive data collection on behavioral and socioeconomic variables to enhance the robustness and applicability of the findings.

### 4.2. Directions for Future Research

Section 4.1 in the manuscript provides a basis for a structured discussion of future research directions.

Intervention studies are essential for moving from observation to action in medical research. They represent clinical studies in which participants are prospectively assigned to groups to receive specific interventions (e.g., behavioral changes) to evaluate their effects on health outcomes. They are crucial for establishing causal relationships and testing the efficacy of proposed solutions. Intervention studies represent the next logical step in determining whether specific interventions can prevent or mitigate adverse maternal and neonatal outcomes. This transition from identifying problems to testing solutions is crucial for achieving clinical impact and informing public health strategies.

Longitudinal studies are invaluable for understanding disease progression, identifying long-term risk factors, and evaluating outcomes over time, especially for chronic conditions. Our study represents a snapshot of data from 2021 to 2023. However, maternal obesity has profound long-term consequences, including intergenerational effects and health outcomes for both the mother and child that extend far beyond the perinatal period. Longitudinal studies are the only way to fully capture these complex, time-dependent relationships, shifting research from immediate pregnancy outcomes to broader societal impacts.

Other structured approaches for future research can provide a comprehensive understanding of complex health issues, ultimately increasing the potential for applying research in practice. These may include the following:

Multicenter studies: Our study was conducted at a single institution, which limits the generalizability of the results. It is proposed that similar studies be conducted in multiple hospitals or regions to increase the generalizability of the findings to a wider population.

Inclusion of broader socioeconomic and behavioral variables: With the current study, we did not have the opportunity to evaluate important confounding factors such as smoking, socioeconomic background, dietary habits, and physical activity, as these data were not available. Future prospective studies should meticulously collect these data to provide a more nuanced understanding of the complex interplay of factors influencing maternal and neonatal outcomes.

Mixed-methods approach: Qualitative research methods (e.g., interviews with pregnant women, healthcare providers) should be integrated with quantitative data to gain a more comprehensive understanding of the barriers to healthy weight management during pregnancy and perceptions of obesity. This can inform the design of more effective interventions.

Interdisciplinary collaboration: Experts from various fields, such as public health, nutrition, exercise physiology, psychology, and health policy, should be involved to develop comprehensive and sustainable interventions.

### 4.3. Practical Recommendations for Health Authorities

I. Education and National Policy:

- National Awareness Campaign: Develop and implement a broad national campaign targeting young women and women of reproductive age regarding the risks of overweight and obesity before and during pregnancy. Emphasize the benefits of a healthy lifestyle (balanced nutrition and physical activity) for optimal pregnancy outcomes.
- Providing Evidence-Based Information: Providing future/current parents with information about the impact of overweight and obesity, as well as the consequences during pregnancy, on the health of pregnant women and newborn babies.
- Educational Programs: Include in-depth modules on healthy eating, physical activity, and reproductive health in school curricula, starting from an early age. Train teachers and school staff to promote healthy habits.
- Communication Channels: Utilize various communication channels—social media, television, radio, print material, and health seminars.
- National Policies: Introduce policies that promote a healthy lifestyle at the national level; for example, regulations on advertising unhealthy foods, support for physical activity initiatives, and access to affordable healthy foods.
- Tax Incentives: Provide tax incentives for products and services that promote healthy eating.

II. Diagnosis and Intervention:

- Unified Screening Protocols: Develop and implement unified protocols for BMI screening for all women of reproductive age, especially when planning pregnancy and during the first examination for confirmed pregnancy. This should also include the assessment of nutritional status and physical activity.
- Specialist Referral: Encourage early referral to specialists. Create multidisciplinary teams (obstetrician–gynecologist, endocrinologist, nutritionist, physiotherapist/physical activity instructor, psychologist) to provide comprehensive care for pregnant women with overweight and obesity. Long-term follow-up of women with high BMI, as well as those with significant GWG.
- Professional Training: Organize regular training and seminars for general practitioners, obstetricians–gynecologists, midwives, and nurses on the latest recommendations for weight management during pregnancy. Emphasize patient motivation skills and provide individualized advice.

III. Scientific Research and Data:

- National Registry: Create and maintain a national registry of pregnant women with overweight and obesity, including data on gestational complications and pregnancy outcomes. This will allow for tracking trends and evaluating the effectiveness of interventions.
- Research Funding: Encourage and finance scientific research aimed at determining the most effective preventive and interventional strategies for weight management in pregnant women.

To achieve all of this, adequate funding is necessary for the implementation of the aforementioned programs and initiatives. By applying these recommendations, health authorities and clinicians can significantly contribute to reducing the prevalence of overweight and obesity among pregnant women in Bulgaria, improving the long-term health of mothers and children.

## 5. Conclusions

In this retrospective cohort study among women who delivered by cesarean section, a significant proportion of them were affected by overweight and obesity. Among women with a high BMI (over 30.00), a higher proportion of hypertensive disorders and preterm births is observed. Data for our country are insufficient, and a more in-depth study of the problem is needed. Conducting prospective and interventional multicenter studies would lead to a deeper understanding of the problem. A better understanding would enable the implementation of more effective prevention and management strategies, ultimately leading to improved outcomes for both mothers and newborns.

## Figures and Tables

**Table 1:** Mothers’ key data.

Characteristics	N (%)	Mean ± SD (Min–Max)
Age	-	29.33 ± 6.39 (14–48)
Parity		
This CS is first	304 (61.54%)	-
This CS is second/third, etc.	190 (38.46%)	-
Complications during pregnancy		
Moderate preeclampsia	62 (12.55%)	-
Severe preeclampsia	14 (2.83%)	-
GDM	13 (2.63%)	-
IUGR	10 (2.02%)	-
Abruptio (ablatio) placentae	4 (0.80%)	-
Fetal macrosomia	4 (0.80%)	-
Comorbidities		
Arterial hypertension	17 (3.44%)	-
Diabetes (pre-pregnancy)	8 (1.62%)	-
Insulin resistance	15 (3.07%)	-
Autoimmune thyroiditis	29 (5.87%)	-
None registered	295 (59.72%)	-
Anthropometric data		
Height in cm	-	164.12 ± 6.18 (146–189)
Weight in kg	-	81.51 ± 16.97 (48–170)
Waist circumference in cm	-	107.77 ± 10.79 (76–147)
Weight gain during pregnancy in kg	-	15.06 ± 5.94 (1–40)
BMI by classes (according to WHO) and average weight gain values in kg		
Normal weight 18.50–24.99	78 (15.79%)	11.72 ± 3.65 (3–25)
Overweight—25.00–29.99	187 (37.85%)	14.75 ± 4.56 (1–26)
Obesity I degree 30.00–34.99	138 (27.94%)	15.77 ± 6.03 (4–30)
Obesity II degree 35.00–39.99	60 (12.14%)	16.62 ± 7.32 (5–36)
Obesity III degree–over 40.00	31 (6.28%)	19.16 ± 9.59 (3–40)

Abbreviations: SD—standard deviation; CS—cesarean section; GDM—gestational diabetes mellitus; IUGR—intrauterine growth restriction; BMI—body mass index.

**Table 2:** Distribution of premature newborns.

Degree of Prematurity	N	%	Mean Weight ± SD (Min–Max)
I-st degree of prematurity	47	74.60	2299.36 ± 150.45 (2000–2490)
II-nd degree of prematurity	12	19.05	1720.00 ± 138.37 (1530–1960)
III-rd degree of prematurity	3	4.76	1323.33 ± 280.06 (1000–1490)
IV-th degree of prematurity	1	1.59	860 (one case)

Abbreviation: SD—standard deviation.

## Data Availability

Restrictions apply to the availability of these data. Data were obtained from University Hospital “St. George”, Plovdiv, and are available from the authors with the permission of University Hospital “St. George”, Plovdiv.

## Abbreviations

The following abbreviations are used in this manuscript:

AH	Arterial hypertension
CS	Cesarean section
BMI	Body mass index
GDM	Gestational diabetes mellitus
IUGR	Intrauterine growth restriction
GWG	Gestational weight gain
